# Case Report: Combination of Pressure Guidewire and Optical Coherence Tomography-Guided Drug-Coated Balloon Revascularization for Renal Artery Fibromuscular Dysplasia

**DOI:** 10.3389/fcvm.2021.773563

**Published:** 2022-01-13

**Authors:** Yuxi Li, Xinyan Wen, Bo Zheng, Ming Chen, Wei Ma, Jianping Li

**Affiliations:** ^1^Department of Cardiology, Peking University First Hospital, Beijing, China; ^2^Institute of Cardiovascular Disease, Peking University First Hospital, Beijing, China; ^3^Hypertension Precision Diagnosis and Treatment Research Center, Peking University First Hospital, Beijing, China

**Keywords:** fibromuscular dysplasia, pressure guidewire, optical coherence tomography, secondary hypertension, drug-coated balloon

## Abstract

Fibromuscular dysplasia (FMD) is the second common cause of renovascular hypertension. With the advent of endovascular therapy, angiography has become a diagnostic gold standard for FMD. Optical coherence tomography (OCT) by reflecting *in vivo* histology may improve diagnostic and classification accuracy. Renal fractional flow reserve (rFFR), measured by pressure guidewire, may distinguish the patients who may benefit from revascularization by identifying physiologically significant stenoses. However, the role of usage of both OCT and rFFR is not well-studied. We herein report a 17-year-old male with renovascular hypertension due to FMD. Angioplasty of drug-coated balloon (DCB) guided by OCT and FFR favorably achieved blood pressure (BP) control. In conclusion, the utility of both OCT and FFR may be useful for the appropriate selection of patients with renal FMD.

## Introduction

Fibromuscular dysplasia (FMD) is a non-atherosclerotic arterial disease, which is characterized by abnormal cellular proliferation and distorted architecture of the arterial wall (1). FMD most commonly affects the renal artery and is the second-most common etiology of renal artery stenosis (RAS) and renovascular hypertension, especially in young patients (2–4). Based on previous studies, the prevalence of FMD was underestimated and has been drawing attention recently.

The diagnosis and classification of renal FDM were mainly based on angiographic characteristics, among which the “string of beads” appearance was the most striking feature (5). However, FMD had been divided into several subtypes according to histopathological characteristics in early studies, which was no longer available since the advent of endovascular therapy (6). Optical coherence tomography (OCT), which can clearly visualize the medial thickening and wavy intimal lining of FMD, demonstrated to be a good tool for FMD diagnosis and evaluation based on the previous reports (7, 8). Furthermore, stenting is generally not indicated in renal FMD, while angioplasty or drug-coated balloon (DCB) revascularization requires precise evaluation and is guided by intravascular imaging such as OCT and intravascular ultrasound (IVUS).

On the other hand, the revascularization criteria of RAS remain debatable. Based on the latest consensus, the pressure gradient could provide further information to identify with hemodynamically significant stenosis, even though, which was validated on studies of atherosclerotic RAS (5). However, the most common methods to measure pressure gradient were based on catheters rather than by pressure guidewire, which was suitable for distal lesions and could provide much more accuracy and detailed information than catheter (9). We report a case of the combination of pressure guidewire and OCT-guided DCB treatment in a young hypertensive patient with renal FMD.

A 17-year-old male who presented with hypertension for 1 month was admitted to the cardiology department, whose ambulatory blood pressure (ABPM) showed an average blood pressure (BP) of 160/98 mm Hg despite anti-hypertensive therapy consisting of bisoprolol and nifedipine. A smoking history for 7 years was noticed. Physical examination was unremarkable, and abdominal bruit and peripheral edema were absent. ABPM showed elevated mean BP of 154/93 mmHg (daytime: 165/105 mmHg, nighttime: 135/72 mmHg). An ECG showed sinus rhythm with left ventricular hypertrophy, and echocardiography revealed a left ventricular ejection fraction (LVEF) of 71.6%. Laboratory examination revealed a normal result of a full blood cell count, renal, and liver function. Since the young age of the patient, without a family history or risk factors for hypertension, investigation for secondary hypertension was first considered. Initial workup noted elevated aldosterone level and plasma renin activity both beyond the upper limit of reference, which posed a strong suspicion of renovascular hypertension. The stenosis of the main branch of the right renal artery was finally revealed after repeated ultrasound examinations. CT angiography was also performed, which demonstrated the stenosis in the initial segment of the anterior segmental artery of the right kidney. Possible involvement of other vascular beds was ruled out with Doppler ultrasound and enhanced CT. The kidney ultrasound and urinalysis of the patient were normal. A radionuclide renal scintigraphy showed mild impairment of the function of the right kidney and normal function of the left kidney. Thyroid-stimulating hormone, the circadian rhythm of cortisol, level of serum catecholamines, and metanephrines were all normal, and other causes for secondary hypertension were ruled out. Autoimmunity screening was negative, which ruled out underlying vasculitis diseases. The lipid profile was normal with low-density lipoprotein cholesterol (LDL-C) of 2.25 mmol/L. Based on these findings, we tentatively diagnosed the patient with renovascular hypertension secondary to RAS, and FMD was highly suspected due to his young age, smoking history, and no further atherosclerosis evidence.

**Figure 1:**
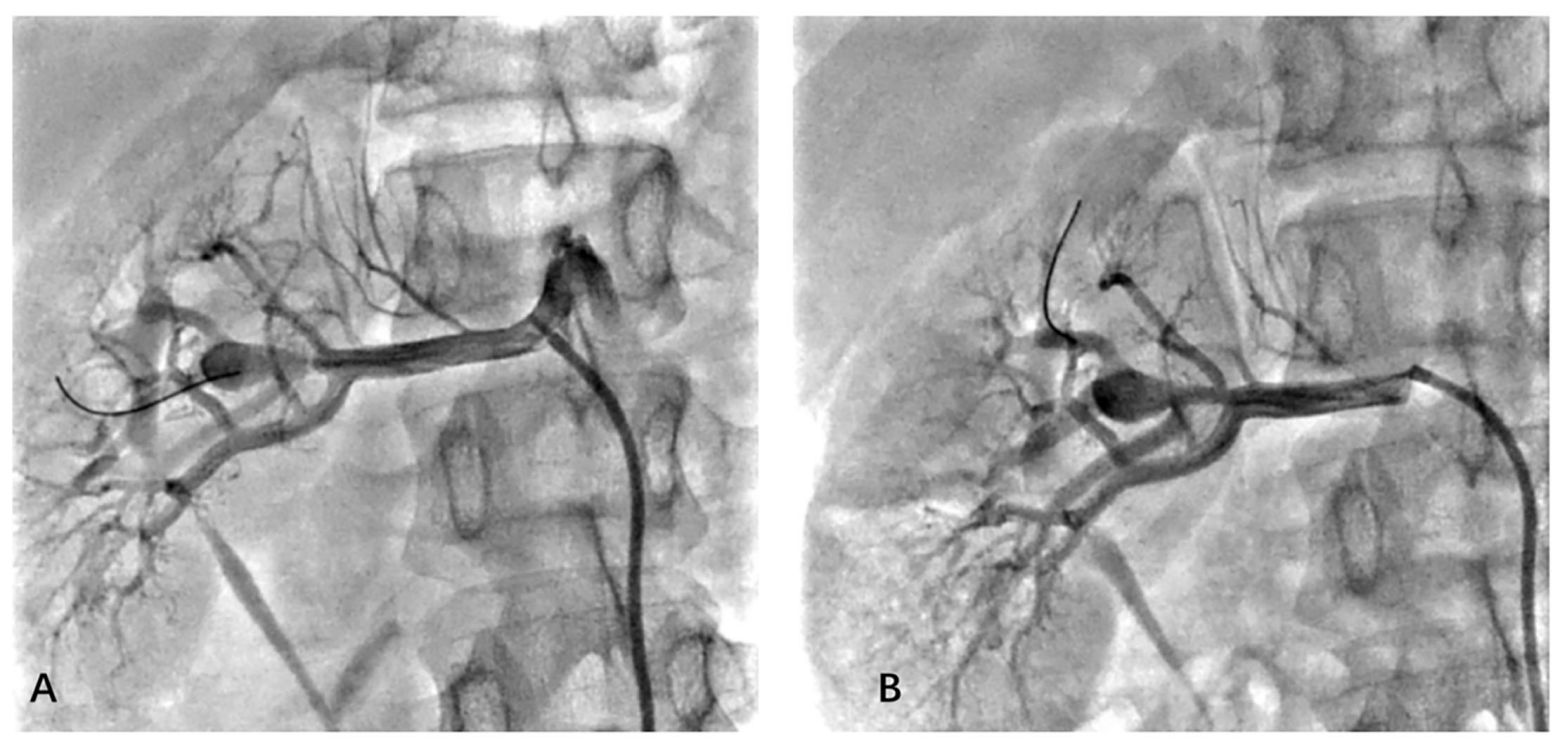
Selective right renal angiography before **(A)** and after **(B)** balloon angioplasty.

**Figure 2:**
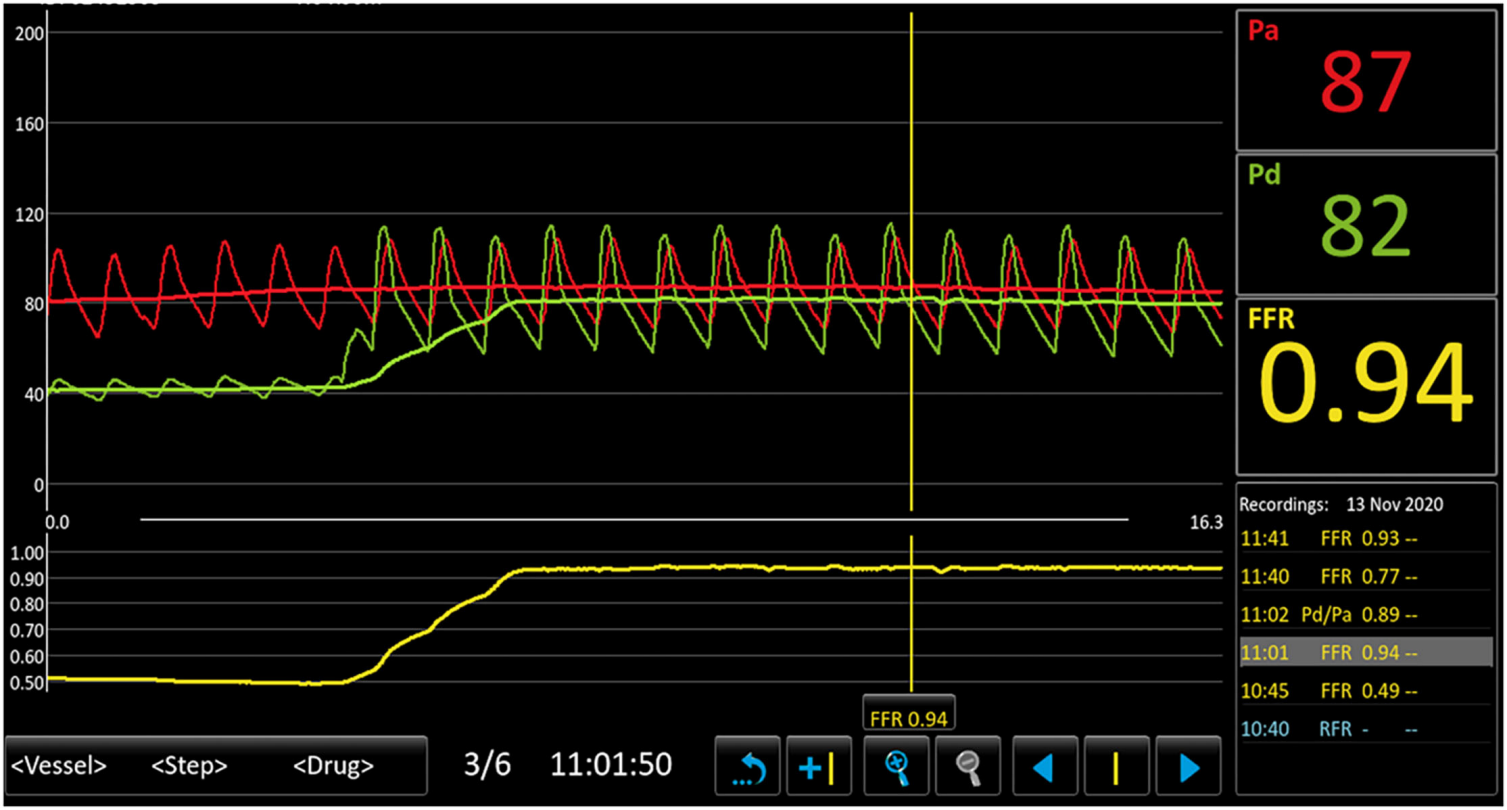
Results of pressure guidewire measurement depicting Pa, Pd, and FFR before and after balloon angioplasty. This figure demonstrates a distal renal artery to proximal renal artery mean pressure gradient of 40 mm Hg and the FFR was 0.49. A significant increase in Pd was found after the pullback of the guidewire. Pa, mean aortic pressure; Pd, pressure distal to the stenosis; FFR, fractional flow reserve.

**Figure 3:**
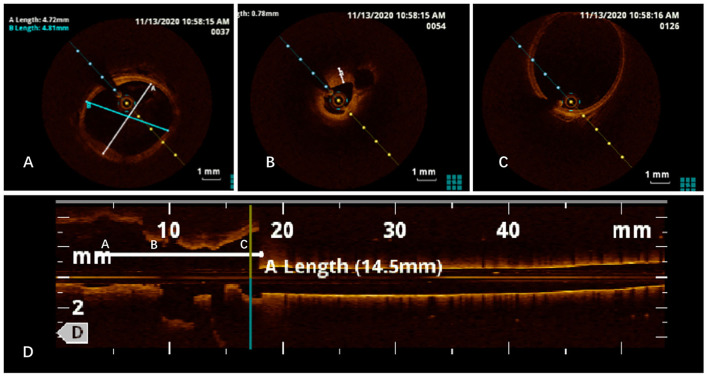
Optical coherence tomography (OCT) imaging revealed normal intimal **(A)**, intimal hyperplasia **(B)**, post-stenotic dilatation **(C)**, and wavy endoluminal surface in the longitudinal reconstruction **(D)**.

To confirm the diagnosis, renal angiography was performed (Figure 1). After insertion of a 6F guiding catheter through the femoral artery, selected angiography showed focal stenosis in the mid-segment of the right renal artery, accompanied by post-stenotic dilatation. A 0.014-inch pressure guidewire (Pressure Guidewire, MN, USA, Abbott), which was advanced across the lesion after equalization, was used to measure the pressure gradient. The value of rFFR (defined as the ratio of mean arterial pressure distal to the stenosis (Pd) to the mean aortic pressure measured from the guiding catheter (Pa), as follows: rFFR = Pd/Pa) was 0.49, and the mean artery pressure gradient at resting was 40 mmHg before the revascularization. A significant increase of Pd was found after the pullback of the guidewire when across the lesions (Figure 2). OCT (C7 XR, MA, USA, Abbott) showed that the minimal lumen area of the renal artery was 1.92 mm^2^ and the wavy endothelium of the right renal artery in longitudinal reconstruction and luminal stenosis due to intimal hyperplasia in cross-sectional imaging, which was compatible with pathognomonic for FMD (Figure 3). In view of difficult-to-control hypertension of short duration with the goal of a cure of hypertension, OCT-guided right renal artery angioplasty with DCB was performed, which resulted in good blood flow across the lesion and no residual trans-lesion pressure gradient (rFFR 0.93, pressure gradient 5 mmHg after revascularization). After the procedure, the BP of the patient decreased to 130–140/80–90 mm Hg with candesartan 4 mg one time a day and was normalized with candesartan 2 mg daily during the next 6 months of follow-up. A smoking cessation education was also provided for the patient.

## Discussion

To the best of our knowledge, this is the first published case combining the pressure guidewire and OCT to guide the endovascular therapy of DCB in renal FMD. FMD is the second common cause of renovascular hypertension, and balloon angioplasty is the revascularization approach of choice for renal artery FMD. With the advancement of intravascular imaging, it has been inaccessible to histological specimens and catheter-based angiography becomes the gold standard modality for the diagnosis of renal artery FMD nowadays (3). Several recently published cases showed that OCT can clearly visualize three layers of renal arteries. Therefore, the utility of OCT in confirming the diagnosis in the angiographically ambiguous lesions, properly classifying FMD, and guiding and optimizing the intervention has been reported (8, 10, 11).

Revascularization for the renal artery FMD is predicated on clinical indicators beyond the simple presence of FMD (3). However, several studies suggested that medial fibroplasia may progress with time (12). The pressure gradient is mandated to evaluate the degree of stenoses, which can predict the BP response after revascularization (13, 14). In addition, a pressure gradient of 10% mean pressure is proposed as a threshold to perform balloon angioplasty, based upon the study of atherosclerotic RAS (5). However, the pressure gradient is affected by multiple factors, such as systemic BP, the state of renal blood flow, and renal microvasculature. There is a lack of evidence from the study of FMD.

Fractional flow reserve is a lesion-specific indicator, which is recommended by guidelines to determine lesion significance and appropriateness for revascularization in coronary artery disease. Subramanian et al. (15) demonstrated that rFFR correlated well with pressure gradient and was a good predictor of lesion severity in the setting of RAS. Mitchell et al. (16) found that rFFR could identify people who are likely to benefit from percutaneous renal revascularization in terms of BP. In contrast, Leesar et al. (14) reported that FFR was not a predictive indicator for BP improvement and Kadziela et al. (17, 18) showed that FFR did not predict BP response and kidney function improvement after renal revascularization. These results are quite opposite to the observations by the investigators mentioned above. The reason for this difference in the observations is not clear; one possible reason may be the method of measuring trans-lesion pressure gradient was based on catheters rather than pressure guidewire. The FFR result could be more precise when measured by a pressure guidewire than a 4f catheter, furthermore, the hyperemic pressure gradient by dopamine could only be measured by a pressure guidewire, and the pressure guidewire was the only method when facing significant stenosis lesions like this case. Moreover, these studies were conducted among patients with atherosclerotic RAS and have not been validated in patients with FMD. The success rate of revascularization among non-medial types of FMD (65%) was lower than those observed in the medial type of FMD (79–100%) (1, 19). These data suggest that the FMD subtype may determine the success rate of the procedure. The combination of OCT and renal FFR may be useful for the appropriate selection of patients with renal hypertension.

In conclusion, the combination of OCT and rFFR may improve the accuracy of diagnosis and classification, gauge the severity of stenosis, identify a patient who may respond to revascularization, and guide the procedure. Further research confirming the efficacy of OCT in the diagnosis and classification of FMD, and the prognostic value of FFR in a different type of renovascular FMD is warranted.

## Data Availability Statement

The original contributions presented in the study are included in the article/supplementary material, further inquiries can be directed to the corresponding author/s.

## Ethics Statement

The studies involving human participants were reviewed and approved by Ethics Committee of Peking University First Hospital. Written informed consent to participate in this study was provided by the participants' legal guardian/next of kin. Written informed consent was obtained from the minor(s)' legal guardian/next of kin for the publication of any potentially identifiable images or data included in this article.

## Author Contributions

YL, BZ, MC, and JL contributed to the clinical treatment of this case. YL and XW contributed to manuscript writing. WM contributed to the review of the manuscript. All authors listed have contributed sufficiently to the project in order to be included as authors and approved the final version of the manuscript for publication.

## Funding

This manuscript was funded by the Youth Clinical Research Project of Peking University First Hospital (No. 2018CR05).

## Conflict of Interest

The authors declare that the research was conducted in the absence of any commercial or financial relationships that could be construed as a potential conflict of interest.

## Publisher's Note

All claims expressed in this article are solely those of the authors and do not necessarily represent those of their affiliated organizations, or those of the publisher, the editors and the reviewers. Any product that may be evaluated in this article, or claim that may be made by its manufacturer, is not guaranteed or endorsed by the publisher.

## References

[1] Slovut DP Olin JW. Fibromuscular dysplasia. N Engl J Med. (2004) 350:1862–71. 10.1056/NEJMra032393 15115832

[2] Nair R Vaqar S. Renovascular Hypertension StatPearls. Treasure Island, FL: StatPearls Publishing Copyright (2021). 31869068

[3] Olin JW Gornik HL Bacharach JM Biller J Fine LJ Gray BH. Fibromuscular dysplasia: state of the science and critical unanswered questions: a scientific statement from the American Heart Association. Circulation. (2014) 129:1048–78. 10.1161/01.cir.0000442577.96802.8c 24548843

[4] Persu A Giavarini A Touze E Januszewicz A Sapoval M Azizi M. European consensus on the diagnosis and management of fibromuscular dysplasia. J Hypertens. (2014) 32:1367–78. 10.1097/HJH.0000000000000213 24842696

[5] Gornik HL Persu A Adlam D Aparicio LS Azizi M Boulanger M. First international consensus on the diagnosis and management of fibromuscular dysplasia. Vasc Med. (2019) 24:164–89. 10.1177/1358863X18821816 30648921

[6] Harrison EG Jr. McCormack LJ. Pathologic classification of renal arterial disease in renovascular hypertension. Mayo Clin Proc. (1971) 46:161–7. 5553126

[7] Otaal PS Batta A Sahoo SK Vijayvergiya R. Overcoming diagnostic dilemma and optimizing intervention with optical coherence tomographic guidance in an angiographically ambiguous renal artery stenosis due to fibromuscular dysplasia. Int Med Case Rep J. (2021) 14:435–41. 10.2147/IMCRJ.S317869 34234576 PMC8254557

[8] Mizutani K Itoh A Sugioka K Komatsu R Naruko T Yoshiyama M. Intravascular findings of fibromuscular dysplasia on optical coherence tomography. J Cardiol Cases. (2015) 12:39–42. 10.1016/j.jccase.2015.03.009 30524536 PMC6262158

[9] Colyer WR Cooper CJ Burket MW Thomas WJ. Utility of a 0.014” pressure-sensing guidewire to assess renal artery translesional systolic pressure gradients. Catheter Cardiovasc Interv. (2003) 59:372–7. 10.1002/ccd.10508 12822163

[10] Kume T Akasaka T Kawamoto T Watanabe N Toyota E Neishi Y. Assessment of coronary intima–media thickness by optical coherence tomography: comparison with intravascular ultrasound. Circ J. (2005) 69:903–7. 10.1253/circj.69.903 16041157

[11] Vijayvergiya R Kanabar K Krishnappa D Kasinadhuni G Sharma A Akasaka T. Optical coherence tomography in varying aetiologies of renal artery stenosis: a case series. Eur Heart J Case Rep. (2019) 3:68. 10.1093/ehjcr/ytz068 31449625 PMC6601173

[12] Goncharenko V Gerlock J Shaff MI Hollifield JW. Progression of renal artery fibromuscular dysplasia in 42 patients as seen on angiography. Radiology. (1981) 139:45–51. 10.1148/radiology.139.1.7208940 7208940

[13] Mangiacapra F Trana C Sarno G Davidavicius G Protasiewicz M Muller O. Translesional pressure gradients to predict blood pressure response after renal artery stenting in patients with renovascular hypertension. Circ Cardiovasc Interv. (2010) 3:537–42. 10.1161/CIRCINTERVENTIONS.110.957704 21078879

[14] Leesar MA Varma J Shapira A Fahsah I Raza ST Elghoul Z. Prediction of hypertension improvement after stenting of renal artery stenosis: comparative accuracy of translesional pressure gradients, intravascular ultrasound, and angiography. J Am College Cardiol. (2009) 53:2363–71. 10.1016/j.jacc.2009.03.031 19539148

[15] Subramanian R White CJ Rosenfield K Bashir R Almagor Y Meerkin D. Renal fractional flow reserve: a hemodynamic evaluation of moderate renal artery stenoses. Catheter Cardiovasc Interv. (2005) 64:480–6. 10.1002/ccd.20318 15789382

[16] Mitchell JA Subramanian R White CJ Soukas PA Almagor Y Stewart RE. Predicting blood pressure improvement in hypertensive patients after renal artery stent placement: renal fractional flow reserve. Catheter Cardiovasc Interv. (2007) 69:685–9. 10.1002/ccd.21095 17351955

[17] Kadziela J Prejbisz A Michalowska I Adamczak M Warchol-Celinska E Pregowska-Chwala B. Relationship between hemodynamic parameters of renal artery stenosis and the changes of kidney function after renal artery stenting in patients with hypertension and preserved renal function. Blood Press. (2015) 24:30–4. 10.3109/08037051.2014.958304 25268986

[18] Kadziela J Januszewicz A Prejbisz A Michalowska I Januszewicz M Florczak E. Prognostic value of renal fractional flow reserve in blood pressure response after renal artery stenting (PREFER study). Cardiol J. (2013) 20:418–22. 10.5603/CJ.2013.0101 23913461

[19] Barrier P Julien A Guillaume C Philippe O Herve R Francis J. Technical and clinical results after percutaneous angioplasty in nonmedial fibromuscular dysplasia: outcome after endovascular management of unifocal renal artery stenoses in 30 patients. Cardiovasc Intervent Radiol. (2010) 33:270–7. 10.1007/s00270-010-9818-x 20165847

